# Bladder Cancer and Urothelial Impairment: The Role of TRPV1 as Potential Drug Target

**DOI:** 10.1155/2014/987149

**Published:** 2014-05-08

**Authors:** Francesco Mistretta, Nicolò Maria Buffi, Giovanni Lughezzani, Giuliana Lista, Alessandro Larcher, Nicola Fossati, Alberto Abrate, Paolo Dell'Oglio, Francesco Montorsi, Giorgio Guazzoni, Massimo Lazzeri

**Affiliations:** Department of Urology, Ospedale San Raffaele, Vita-Salute San Raffaele University, Via Stamira D'Ancona 20, 20127 Milan, Italy

## Abstract

Urothelium, in addition to its primary function of barrier, is now understood to act as a complex system of cell communication that exhibits specialized sensory properties in the regulation of physiological or pathological stimuli. Furthermore, it has been hypothesized that bladder inflammation and neoplastic cell growth, the two most representative pathological conditions of the lower urinary tract, may arise from a primary defective urothelial lining. Transient receptor potential vanilloid channel 1 (TRPV1), a receptor widely distributed in lower urinary tract structures and involved in the physiological micturition reflex, was described to have a pathophysiological role in inflammatory conditions and in the genesis and development of urothelial cancer. In our opinion new compounds, such as curcumin, the major component of turmeric *Curcuma longa*, reported to potentiate the effects of the chemotherapeutic agents used in the management of recurrent urothelial cancer in vitro and also identified as one of several compounds to own the vanillyl structure required to work like a TRPV1 agonist, could be thought as complementary in the clinical management of both the recurrences and the inflammatory effects caused by the endoscopic resection or intravesical chemotherapy administration or could be combined with adjuvant agents to potentiate their antitumoral effect.

## 1. Introduction

Inflammation and neoplastic cell growth represent the main two pathologies of the bladder and they have a huge impact on patient's quantity and life-span.

The urothelium has been recognized to be more than a simple barrier separating the luminal contents from the inner layers of the urinary tract. It works with suburothelium as a functional unit, which responds to external stresses by the release of modulator agents that regulate the activity of not only the nearby afferent nerves but also of the underlying smooth muscle and urothelial stem cells. In particular, it may act as an efficient chemomechanosensor, the “afferent function,” and at the same time, it is able to synthesize and release, into suburothelium layer, molecules involved in the bladder storage/voiding activity, the “efferent function.” Furthermore, urothelium may protect the basal cells from toxins or other substances capable of activating a pathological cell growth.

Since the early 90s, investigators focused their basic science and clinical research on the expression, function, and clinical application of a subset of capsaicin-sensitive primary sensory afferents of the lower urinary tract (LUT) [1–7].

Both the upper and LUT are densely innervated by capsaicin-sensitive primary afferent neurons in a number of species including humans [8]. Early pharmacological studies revealed that capsaicin-sensitive, C type, bladder fibers play a role in micturition reflex and it was shown that capsaicin sensitive nerves exhibit both a sensory (afferent) and an “efferent” function, which is determined by the release of peptides including tachykinins, substance P (SP) and calcitonin gene-related peptide (CGRP) [9]. The sensory function includes the regulation of the micturition threshold and the perception of pain from the urinary bladder, while the efferent function controls nervous tissue excitability, smooth muscle contractility and plasma protein extravasation (neurogenic inflammation). The discovery of specific binding sites for capsaicin in several tissues and organs, including the rat urinary bladder [9], initiated a rush that ended up with the cloning of the vanilloid receptor [10], presently known as TRPV1 (transient receptor potential vanilloid subfamily 1).

In the lower urinary tract, TRPV1 expression is now firmly documented not only in a large subpopulation of nerve fibers but also in nonneuronal tissues. Knowledge about the presumable function of TRPV1 also evolved rapidly. From a receptor initially considered as an integrator of thermal and chemical noxious stimuli, TRPV1 is emerging as a possible regulator of bladder reflex activity and cell differentiation. These findings, together with the promising clinical applications of TRPV1 targeting in the LUT, justified our interest in the distribution and function of capsaicinoids and their receptors in normal and pathological conditions. Recently it has been demonstrated that capsaicin and other vanilloids, which are linked to TRP receptors, may promote cellular death [11] and inhibit the growth of normal and neoplastic cells by apoptosis induction [12–15].

In this paper, we report the documented role of TRPV1 in the transitional cell carcinoma (TCC) of human bladder in presence of urothelium impairment and explore the opportunity of considering TRPV1 as a drug target.

## 2. Bladder Cancer

Bladder cancer is the second most common genitourinary cancer in the United States, overall the fourth most frequent cancer in men and the ninth in women, with 72,570 new cases (54,610 men and 17,960 women) and 15,210 deaths both in men and women predicted to occur in 2013 [16].

Usually, bladder cancer is distinguished in muscle invasive and non-muscle-invasive urothelial carcinoma of the bladder (NMIBC). Due to its pathophysiological characteristics and the worldwide assessed management workup, NMIBC has a high probability to recur with a low progression and metastasis rate. For the management of the noninvasive bladder cancer EAU, FICBT, NCCN, and AUA guidelines agree about the importance of endoscopic resection in all patients and the benefit of adjuvant intravesical therapy [17].

The benefits of a single, early, intravesical instillation of mitomycin C after transurethral bladder resection in patients with low risk NMIBC have been investigated. In 1999, Solsona et al. showed how mitomycin C instillation at 24-month follow-up significantly increased the recurrence free interval, but the result could not be reproduced at long-term follow-up. More recently, a single center study described a significant reduction in risk of both early and late recurrences [18]. In 2013, an updated meta-analysis confirmed that intravesical administration of chemotherapy after transurethral of NMIBC prolongs recurrence free interval and reduces early recurrences [19].

However, mitomycin C was seen to own several side effects and in particular after transurethral resection it has been described to delay the healing of the mucosa at resection sites both in animal and human [20, 21]. Urologists remained unconvinced that the benefits of MMC (immediate + maintenance), including a 30% relative reduction in the recurrence of a nonlethal disease, outweigh the potential harms, for example, cystitis, which can occasionally be severe and irreversible [22, 23].

In our opinion, to delay the recurrence time, from the endoscopic treatment procedure, of patients affected by NMIBC is of outstanding importance. In 2004, Sylvester et al. demonstrated, with a meta-analysis of seven randomized clinical trials, that single intravesical chemotherapy (IVC) following transurethral resection reduces recurrences [23]. Despite this, the adoption of this practice has been modest and a recent analysis described the rate of perioperatively intravesical administration of chemotherapy in as few as 0.3% to 3.2% cases [24].

Recently, the consideration that an urothelial disfunction could be the basis of benign bladder pathology: barrier disfunction in pathological bacteria urinary infections, increased permeability, decreased E-cadherin content, and an increased number of apoptotic urothelial cells in interstitial cystitis (painful bladder syndrome), has grown up. Sensory function impairment due to stones or neoplasms can cause changes to the urothelium resulting in bladder overactivity and urge incontinence, or in underactive bladder in case of outlet obstruction [25]. Similarly, an urothelial disfunction could be supposed to be linked with the development of bladder cancer. Based on such observation, some compounds that are able to repair the urothelium defect are now entering the clinical practice for the treatment of urothelial dysfunction. We could point out hyaluronic acid for the therapy of not malignant bladder conditions, such as bladder pain syndrome, interstitial cystitis, or recurrent urinary tract infections [26], or in combination with paclitaxel for the treatment of bladder bacillus Calmette-Guérin refractory carcinoma in situ [27].

## 3. Urothelium Impairment

The urothelium is the most superficial layer of the urinary tract that separates the lumen from underlying tissues of the wall. Formed by three layers, a basal, an intermediate, and a superficial or apical layer was composed of large hexagonal cells known as “umbrella cells” [28]; there are now strong evidences that the urinary bladder urothelium exhibits specialized sensory properties and plays a role in the detection and transmission of both physiological and mechanical stimuli, such as luminal pressure, urine composition, and nociceptive stimuli, beyond acting as an effective barrier [29]. Bladder's barrier function is conferred by a mucin layer formed by sulphated polysaccharide glycosaminoglycan (GAG), which covers the cellular apical surface. The mucin layer acts as a nonspecific antiadherence factor and as a defence mechanism against infection and irritants [30], but several agents, such as chronic bacterial infections, autoimmune diseases, chemotherapeutic agents, or external sources (e.g., radiation exposure), can lead to urothelial damage and loss of the GAG function [31].

There is a wide consensus that many clinical conditions may arise from a primary defective urothelial lining [32] and in particular from a GAG injury. This injury induces a loss of the watertight function and leads to an infiltration of normal and abnormal constituents of urine through the lesion causing a failure in the healing process and producing chronic bladder epithelial damage and neurogenic inflammation [33]. In a randomised placebo-controlled trial, it has been shown that, restoring the GAG layer with intravesical administration of a combination of hyaluronic acid and chondroitin sulphate, in women with a recurrent urinary tract infection (UTI), the UTIs rate could be reduced without causing severe side effects while improving quality of life over a period of a year [34].

As mentioned previously, bladder urothelium acts as a specialized sensory tissue mediating both afferent and efferent signals through a flourishing subset of receptors and mediators. Receptors for purines [35], noradrenaline [36], bradykinin [37], and acetylcholine [38, 39] and several transient receptor potential (TRP) channels (TRPV1, TRPV2, TRPV4, TRPM8, TRPA1) [40–43] are expressed on the membranes of urothelial cells. From a neural point of view, an urothelial damage and the loss of the GAG function lead, in the suburothelium, to the activation of a subset of unmyelinated C fibres selectively sensitive to capsaicin. These unmyelinated C fibres serve as primary afferents in the regulation of micturition reflex and pain sensation and activation of visceral reflex but are even involved, through their efferent function, in the regulation of the lower urinary tract influencing the smooth muscle contraction [44], immune cell migration, mast cells degranulation, and neurogenic inflammation, thus playing a role in bladder inflammation [45].

These notions, added to the description of a decrease in both rate of contraction and bladder hyperreflexia in cyclophosphamide-inflamed rat urinary bladders after administration of Capsazepine, a selective antagonist for TRPV1 [46], lead to speculation about a role of this family of sensory receptor in the treatment of cystitis-induced hyperalgesia, through targeting their activity on C fibres.

Furthermore, the prolonged GAG defect persistence leads to a chronic stimulation of suburothelial tissues, which results in the allodynia caused by a visceral hypersensitivity of bladder C-fibre nociceptors, and in molecular changes, such as altered expression of transcription factors or activation of cellular inflammatory pathways (e.g., NF-*κ*B related), producing an increase in frequency, urgency, nocturia, and chronic pain, characteristic symptoms of cystitis [47, 48].

According to these basics and to the more recent studies [26, 49], the early repair of the mucin layer by intravesical administration of mucopolysaccarides should be obtained to avoid the chronic evolution of the bladder inflammatory pathologies. A chronic defect of GAGs might allow irritants to reach the basal cells and to determine an anomalous cell growth.

## 4. Transient Receptor Potential Vanilloid 1

Transient receptor potential (TRP) superfamily of ion channels was first cloned from the visual system of* Drosophila *at the end of the 1980s [50].

In mammalian, the 28 TRP superfamily members are expressed in the plasma membrane of several cell types, principally mediating calcium and sodium inward membrane currents [51].

TRP proteins were first divided into three main subfamilies: the canonical or classical TRP proteins (TRPC), the vanilloid receptor related TRP proteins (TRPV), and the melastatin-related TRP proteins (TRPM). Later, the mucolipins (TRPML), the olycystins (TRPP), and the ankyrin transmembrane protein 1 (TRPA1) subfamilies have been included [40].

Numerous members of TRP superfamily of ion channels are expressed in the LUT. TRP expression in the mouse bladder was described showing the presence of 27 TRP family members mRNA in whole bladder tissue [52]. A pivotal role of the thermosensitive TRPs TRPV1, TRPM8, and TRPA1 and of TRPV2 and TRPV4, in normal and pathological LUT function, has been established, mainly as sensors of stretch or chemical irritation [53, 54], but information regarding the functional significance of others TRP proteins in the LUT still remains limited.

In 1993, Szallasi et al. characterized binding sites for capsaicin in rat urinary bladder [55], while in 1997 Caterina et al. cloned the vanilloid receptor presently known as TRPV1 [10], a nonselective, highly Ca^2+^-permeable cationic channel.

This channel is widely distributed in LUT structures [15] and, although there are few solid data, it has been reported that in some species, for example, mouse, the TRPV1 channel is expressed not only in bladder neurones, but also in urothelial cells' membranes [56, 57].

Firstly, TRPV1 used to be considered as an integrator of thermal and chemical noxious stimuli, but evidences led it to be thought as a possible regulator of bladder reflex activity and cell differentiation [58].

The role of TRPV1 in bladder function is well established and basic scientific evidence supports TRPV1 action in the regulation of the frequency of bladder reflex contractions, even in chronically inflamed rat urinary bladders [46].

TRPV1 channels in the suburothelium are needed for normal excitability of low-threshold bladder fibres. In TRPV1-knockout mice low-threshold response has been seen attenuated compared with their wild type TRPV1 littermates, whereas high-threshold sensitivity was unchanged [59]. Furthermore, the knockout of TRPV1 in mice led to intermicturition spotting, suggested by an increase in the frequency of nonvoiding contractions in cystometrograms, but it does not affect normal micturition [53].

Maggi et al. described in humans a functional modulation of capsaicin sensitive C-fibers on the micturition reflex. Moreover, after the intravesical administration of capsaicin, authors observed a reduction of the first desire to void, bladder capacity and pressure threshold for micturition, and an attenuation of symptoms of hypersensitive LUT disorders. Moreover, TRPV1 activation generates a burning pain sensation, similar to that reported by CP/CPPS patients during micturition and ejaculation [60].

Although both capsaicin and resiniferatoxin (RTX) have been tested by intravesical administration to reduce pain in patients with painful bladder syndromes, including interstitial cystitis [6, 60, 61], the first large randomized clinical trial testing several concentrations of RTX, compared against placebo, did not detect any advantage in the use of the compound [62].

Moreover, the expression of TRPV1 in terminal nerves ending near to the mucosa and to the smooth muscle cells could indicate its involvement in maintaining the tone of the muscular layer [51], and the relationship between the capsaicin selective activation of bladder TRPV1 and the increase of dieresis and natriuresis supported the hypothesis of a TRPV1 involvement in fluid homeostasis [2].

It is well described how malignant cell transformation is often accompanied by changes in ion channel expression [63] and among them several members of the TRP family Ca^2+^ and Na^+^-permeable channels show altered expression in cancer cells.

To date, most changes involving TRP proteins do not involve mutations in the TRP gene but rather an increase or a decrease in the wild type TRP protein levels of expression, depending on the stage of the cancer. It is not yet possible to say whether these changes in TRP expression are central steps in the progression of the cancer or are secondary to other cellular modifications. Irrespective of the answer to this question, several TRP proteins have been shown in the last years to be valuable markers in predicting the progress of urological cancers and have been considered potential targets for pharmaceutical treatment [64–66].

Recently, the expression of TRPV1 has been described associated with urothelial cancers of human bladder [67].

Capsaicin, the major TRPV1 agonist, has been shown to inhibit in vivo and in vitro cancer growth and progression and to induce apoptosis of different cancer cells [68–70].

An antimigration activity of capsaicin on highly metastatic B16-F10 melanoma cells was investigated. A significant inhibition in the migration of melanoma neoplastic cells, effect correlated with the downregulation of phosphatidylinositol 3-kinase (PI3-K) and of Akt, PI3-K downstream target were described. Moreover in the same study, capsaicin was found to significantly inhibit Rac1 activity in a pull-down assay [71].

The genetic deficiency of TRPV1 was described to induce a higher incidence and number of tumors in the distal colon and an accentuated development of colonic adenomas, in mice affected by colitis-associated cancer and in APCMin−/+ model of spontaneous colon cancer, respectively [72].

In bladder urothelium, Lazzeri et al. firstly demonstrated how the expression of TRPV1 decreases from normal urothelium to transitional cell carcinoma (TCC) of human bladder. From specimens, obtained by multiple cold cup and full-thickness biopsy during open surgery, they showed a reduced labelling intensity after immunohistochemistry in superficial TCC a very light labelling was occasionally detected in the muscle invasive TCC from scattered superficial cells, and no labeling was present in the basal cells and in those that had invaded the adventitia. Similarly, in the Western Blot, which in the controls recognized two thick stained bands, in all superficial TCCs the two bands were similar to control ones, whereas they were very thin in muscle invasive and no band was detected in the patients staged as pT4 [73].

More recently, Amantini et al. displayed a marked decrease or absence of TRPV1 labelling in urothelial cancer specimens proportionally to differentiation levels decrease after a quantitative real-time PCR and that TRPV1 mRNA level was highly expressed in low-grade cancers, whereas its expression, confirming the previous results, was reduced in high-grade tumors or in advanced stage invasive pathologies. In the same study, the treatment of low-grade RT4 human urothelial cell carcinoma with capsaicin at 100 *μ*M dose induced a TRPV1-dependent G0/G1 cell cycle arrest and apoptosis, effect that was seen associated with the transcription of proapoptotic genes including Fas/CD95, Bcl-2, and caspases, and the activation of the DNA damage response pathway [74].

On the other hand, attention has to be paid to the Capsaicin property to exhibit tumor-promoting effects, in a receptor-dependent manner, in particular in cancer strain cells lacking TRPV1 receptor, where the transfection with the TRPV1 cDNA leads to an increase in capsaicin-mediated calcium level, growth inhibition, apoptosis, and capsaicin-induced migration regression, suggesting that the TRPV1 plays an inhibitory role in urothelial cancer invasion and metastasis [75].

However, it is necessary to recognize that the mechanism of action of agonists such as capsaicin may be independent by TRPV1 activation. An example is the aforementioned work of Shin et al. on B16-F10 melanoma cells, where the authors described how capsaicin could have a role in the regulation of intracellular pathways independently from TRPV1 activity [71]. Other studies suggested an inhibition of migration induced by capsaicin without an involvement of TRPV1.

In 2002, Surh indicated that capsaicin could mediate apoptosis in human skin cancer cells through the inhibition of mitochondrial and plasma membrane electron transport systems inducing an excessive generation of reactive oxygen species [76]. In the same way, an increase in the reactive oxygen species after capsaicin administration was confirmed in 2005 by Qiao et al. [77].

Recently, Gonzales et al. demonstrated that, in vitro and in mouse xenografts, the local delivery of capsazepine decreases cellular duplication rate and reverses the growth of oral squamous carcinoma cells, inducing the production of reactive oxygen species and apoptosis, and mediating these actions independently from TRPV1 activation. This data was confirmed by calcium imaging technique, which showed how TRPV1, even if present, did not respond to capsaicin (alone or in combination with capsazepine) activation at noncytotoxic concentrations in all cancer cell lines, whereas a significant calcium influx was described, in positive controls, after ionomycin (nonselective cation channel agonist) administration. Moreover, they described that at equal concentration capsazepine is more effective at inhibiting cell viability than capsaicin, without adverse effects on nonmalignant tissues, after in vitro and in vivo administration of the TRPV1 antagonist [78].

All the data showed lead to speculation about a possible clinical involvement for the TRPV1, not only for the treatment of bladder urothelial inflammatory condition or in pain reduction in patients with painful bladder syndromes, but even in the management of tumoral conditions.

The in vivo and in vitro cancer growth and progression inhibition, the induction of apoptosis, and the antimigration activity of capsaicin in tumor of other tissues, such as gastric cancer and glioma, and the studies of Lazzeri and Amantini's groups suggest a theoretical role of targeting the TRPV1 for clinical management of urothelial cancer.

In particular, a modulation of TRPV1 activity could be interestingly investigated in the management of NMIBC after a transurethral resection for both the anti-inflammatory and cancer recurrence rate decreasing possible features.

## 5. Curcumin

Curcumin [diferuloylmethane, 1,7-bis-(4-hydroxy-3-methoxyphenyl)-1,6-heptadiene-3,5-dione] is an active principle held inside in the rhizome of* Curcuma* species widely used as a yellow coloring and flavoring agent in food [79].

Its pharmacological benefits have been appreciated since ancient times in particular for the antioxidant and anti-inflammatory properties.

Curcumin's metabolism is explicated by the endogenous reductase system that reduces it to dihydrocurcumin, tetrahydrocurcumin (THC). and trace of hexahydrocurcumin, in a stepwise manner, to be lately glucuronidated by the uridine 5′-diphospho (UDP)-glucuronosyltransferase [80]. Furthermore, some studies have described a higher antioxidant activity [81, 82], and in some models a higher preventive effect on carcinogenesis [83] of tetrahydrocurcumin was compared to curcumin.

Several studies have shown that curcumin induces apoptosis, via deactivation of nuclear factor-kappa B (NF-*κ*B), and its regulated gene products, in addition to suppression of cell proliferation, invasion, and angiogenesis. Curcumin was also found to suppress several inflammatory cytokines such as tumor necrosis factor-alpha (TNF-*α*), interleukins (IL-1, -1b, -6, and -8), and cyclooxygenase- 2 (COX-2) [84].

Shehzad et al. described the main roles that curcumin may perform in inflammatory pathways (the most important of them are summarized in Table 1) and in the management of the related chronic inflammatory diseases [85].

Cheng et al. demonstrated, in urinary bladder isolated from Wistar rats, that curcumin has an ability to activate M1-mAChR for increase of glucose uptake in muscle. Believed as the most common subtype of muscarinic receptor in skeletal muscle, mAChR has been introduced as an important factor in the regulation of muscle tone.

In the same study, Cheng's group found that curcumin caused a concentration-dependent increase of muscle tone in urinary bladder isolated from Wistar rats. This action was inhibited by pirenzepine at concentration high enough to block M1-mAChR [86].

Due to the pleiotropic beneficial effects of curcumin in inflammatory disease, its role in urothelial pathologies has been speculated.

Though not well established, the main field investigated is the curcumin preventive effect in the development of hemorrhagic cystitis after cyclophosphamide administration.

The prior administration of curcumin before cyclophosphamide challenge, possibly through modulating the release of inflammatory endocoids, was shown to improve all the biochemical and histologic alterations induced by the cytotoxicity, ameliorates the energy status, and restores the oxidant/antioxidant balance [87].

Feasibility and curative effects of an intravesical treatment for cystitis glandularis, a metaplastic alteration of the urothelium in the urinary bladder due to persistent infection, calculi, bladder exstrophy, outlet obstruction, or even tumor, were, respectively, explored and displayed administrating curcumin in 14 patients, diagnosed with the pathology, which remained symptomatic after the state-of-the-art designed primary treatments [88].

Curcumin controls cell proliferation and cycle progression via the modulation of enzymes, growth factors and their receptors, cytokines and various kinase proteins activities. A potential therapeutic involvement has been discussed for pulmonary, digestive system, reproductive system, breast, hematological, thymic, bone, and brain tumors.

Furthermore, studies have shown how not only curcumin, but also its analogues 3,5-Bis(2-fluorobenzylidene)-4-piperidone (EF24) and 3,5-Bis(2-pyridinyl-methylidene)-4-piperidone (EF31), a more potent inhibitor of NF-*κ*B activity than either EF24 or curcumin, exhibit both anti-inflammatory and anticancer activities [89].

In the urological field, curcumin seems to have a role in the management of prostate, kidney, and urothelial bladder cancer regulating cell survival, proliferation, invasion, and angiogenesis (Table 2).

In prostate, curcumin induces apoptosis in androgen-dependent (LNCap) and androgen-independent (DU15) prostate cancer cell lines [90], downregulating antiapoptotic genes, such as Bcl2 and Bcl-xL, and inducing procaspase-3 and 8. Curcumin also inhibits the prostate specific antigen and decreases the expression of AP-1, cyclin D1, NF-*κ*B, cAMP response element-binding (CREB), EGFR tyrosine kinase, and the activities of androgen receptor-dependent NKX3.1 [91]. Furthermore, a decrease of cell proliferation, colony formation, and cell motility and an enhancement of cell aggregation through the activation of protein kinase D1 have been described, which in turn inhibits nuclear b-catenin transcription activity [92]. Herbal preparations based upon curcumin extracts were given to the HGPIN patients three times per day for 18 months. The 18-month biopsy revealed no markers of HGPIN and a reduction in NF-*κ*B and C-reactive protein [93].

In human, bladder cancer cells studies have shown that curcumin induces apoptosis downregulating Bcl2 and increasing the levels of Bax and p53, and moreover it inhibits the development of urothelial tumors in a rat bladder carcinogenesis model [94]. Other effects described are the downregulation of VEGF and VEGF receptor 1 (VEGFR1) and the inhibition of NF-*κ*B and cyclin D1 [95]. Furthermore, it has been described that intravesical injection of curcumin can inhibit bladder cancer in female C57BL/6 mice implanted with MB49 bladder cancer cells [96].

Tharakan et al. described that curcumin potentiates the apoptotic effects of gemcitabine against human bladder cancer, where curcumin also suppresses the cell survival transcription factor NF-*κ*B activated by gemcitabine. Moreover, in orthotopic mouse model curcumin alone significantly reduced the bladder tumor volume and decreased the proliferation marker Ki-67 and microvessel density, but maximum reduction was observed when curcumin was used in combination with gemcitabine. At least, as just described in other studies, they confirmed how curcumin abolishes the constitutive activation of NF-*κ*B in the tumor tissue; decreases cyclin D1, VEGF, COX-2, c-myc, and Bcl-2 expression in the bladder cancer tissue; and induces apoptosis [97].

At least, curcumin has the same vanilloid ring as capsaicin, a particular structure considered necessary for the activation of the TRPV1 receptor [98], suggesting that TRPV1 is a plausible target for the pharmacological action of curcumin.

Several studies have investigated the role of curcumin in the thermal and pain regulatory patterns involving the TRPV1. It was shown that curcumin attenuates thermal hyperalgesia in diabetic neuropathic pain [99], produces an antihyperalgesic effect in a formalin-induced orofacial pain model in rats [100], and ameliorates the severity of damage in dinitrobenzene sulphonic-acid-induced colitis, possibly by acting as a TRPV1 agonist [98]. At least, curcumin reduced capsaicin-induced currents in a dose-dependent manner in both trigeminal ganglion neurons; thus, the antagonism of TRPV1 may provide a potential mechanism underlying the antihyperalgesic effect of curcumin [101].

## 6. Clinical Perspectives

The genesis of neoplastic lesions of urothelial epithelium, in particular of bladder urothelium, recognizes different causes and the major risk factors could be divided into inherited or acquired. The most important risk factor is undoubtedly the habit of smoking, but even the work-related and environmental conditions play an important role.

Among medical conditions, chronic inflammation, chronic urinary retention, and upper tract dilation, which cause the increase of urothelial exposure to carcinogens, are the most pathological features involved in the carcinogenesis.

It has been hypothesized that the inflammatory condition linked to the disruption of the urothelial layer could be involved in the processes of cancer development as seen in other tumoral conditions (e.g., colorectal cancer [102]).

In this context, agents like TRP channel ligands, involved in functional and pathological pathways, could play an important role.

The vanilloid receptor TRPV1, owning a role in the modulation of urothelial inflammatory condition, could be thought as an interesting factor in the management or in the prevention of neoplastic pathologies.

In particular, its targeting could be conjectured in case of NMIBC after a transurethral resection where its characteristic of anti-inflammatory agent could be helpful for the epithelium healing or in the management of patients during the postoperative time.

Moreover, the property of inducing apoptosis and the antimigration activity makes TRPV1 an interesting target in the handling of recurrences.

Curcumin, the major component of turmeric* Curcuma longa*, has been described to own benefic effects in pathological pathways common of both inflammation and carcinogenesis.

Its applications for pathologies involving urothelium disruption such as cystitis glandularis or hemorrhagic cystitis cyclophosphamide-induced have been successfully investigated. In a like manner, its intrinsic property in cell survival and angiogenesis regulation has been shown in several tumor tissues including bladder cancer, where in particular an increase of apoptotic effect has been described after its association with Gemcitabine.

Moreover, owning a vanilloid ring, curcumin might be able to activate the TRPV1 receptor. The combination of the intrinsic properties of curcumin in association with the capacity of acting on TRPV1 could make this compound a very interesting agent in the management of urothelial dysfunctions. However, this hypothesis needs further studies to be confirmed.

In this context new compounds, such as curcumin, could be complementarily used in the clinical practice to manage the recurrences and soothe the inflammatory effect of transurethral resection or intravesical chemotherapy administration, or in combination with the chemotherapies to potentiate the antitumor effect.

## Figures and Tables

**Table 1:** Main inflammatory pathways influenced by curcumin activity.

Chronic disease	Major mediators involved
Arthritis	COX, MMPs, STAT3, NF-κB
Scleroderma	MAP kinase, NF-κB, TGIF
Psoriasis	STAT3, NF-κB, Bcl-xL, IAPs
Allergies and asthma	MAP kinase, NF-κB
Diabetes	PPAR-*γ*, NOS
Obesity	TNF-*α*, IL-6, Wint/*β*-catenin
Neuropathies	IL-1*β*, VEGF, NF-κB, TNF-*α*, NO, LRRK2
Cardiopathies	Bcl-2, IL-6, Caspase, NF-κB, TNF-*α*
Renal ischemia	HSP-70, MAP kinase, NF-κB, TNF-*α*

**Table 2:** Cancer regulator factors influenced by curcumin activity in urological neoplasia.

Urological cancers	Major mediators involved
Prostate cancer	EGFR, AP-1, cyclin D1, NF-κB, CREB
Kidney cancer	Bcl_2_, Bcl-xL, ROS, Akt, TRAIL, IAP
Bladder cancer	Bcl_2_, AP-1, cyclin D1, VEGF, NF-κB

## References

[1] Lecci A, Giuliani S, Lazzeri M, Benaim G, Turini D, Maggi CA (1994). The behavioral response induced by intravesical instillation of capsaicin in rats is mediated by pudendal urethral sensory fibers. *Life Sciences*.

[2] Lazzeri M, Barbanti G, Beneforti P (1995). Vesical-renal reflex: diuresis and natriuresis activated by intravesical capsaicin. *Scandinavian Journal of Urology and Nephrology*.

[3] Lazzeri M, Beneforti P, Benaim G, Maggi CA, Lecci A, Turini D (1996). Intravesical capsaicin for treatment of severe bladder pain: a randomized placebo controlled study. *Journal of Urology*.

[4] Giuliani S, Patacchini R, Lazzeri M (1996). Effect of several bicyclic peptide and cyclic pseudopeptide tachykinin NK2 receptor antagonists in the human isolated urinary bladder. *Journal of Autonomic Pharmacology*.

[5] Lazzeri M, Beneforti P, Turini D (1997). Urodynamic effects of intravesical resiniferatoxin in humans: preliminary results in stable and unstable detrusor. *Journal of Urology*.

[6] Lazzeri M, Beneforti P, Spinelli M, Zanollo A, Barbagli G, Turini D (2000). Intravesical resiniferatoxin for the treatment of hypersensitive disorder: a randomized placebo controlled study. *Journal of Urology*.

[7] Lazzeri M, Spinelli M, Zanollo A, Turini D (2004). Intravesical vanilloids and neurogenic incontinence: ten years experience. *Urologia Internationalis*.

[8] Lecci A, Maggi CA (2001). Tachykinins as modulators of the micturition reflex in the central and peripheral nervous system. *Regulatory Peptides*.

[9] Maggi CA (1990). The dual function of capsaicin-sensitive sensory nerves in the bladder and urethra. *Ciba Foundation symposium*.

[10] Caterina MJ, Schumacher MA, Tominaga M, Rosen TA, Levine JD, Julius D (1997). The capsaicin receptor: a heat-activated ion channel in the pain pathway. *Nature*.

[11] Hiura A, Nakae Y, Nakagawa H (2002). Cell death of primary afferent nerve cells in neonatal mice treated with capsaicin. *Anatomical Science International*.

[12] Lee YS, Nam DH, Kim J-A (2000). Induction of apoptosis by capsaicin in A172 human glioblastoma cells. *Cancer Letters*.

[13] Macho A, Calzado MA, Muñoz-Blanco J (1999). Selective induction of apoptosis by capsaicin in transformed cells: the role of reactive oxygen species and calcium. *Cell Death and Differentiation*.

[14] Jung M-Y, Kang H-J, Moon A (2001). Capsaicin-induced apoptosis in SK-Hep-1 hepatocarcinoma cells involves Bcl-2 downregulation and caspase-3 activation. *Cancer Letters*.

[15] Jambrina E, Alonso R, Alcalde M (2003). Calcium influx through receptor-operated channel induces mitochondria-triggered paraptotic cell death. *Journal of Biological Chemistry*.

[16] Siegel R, Naishadham D, Jemal A (2013). Cancer statistics, 2013. *CA Cancer Journal for Clinicians*.

[17] Brausi M, Witjes JA, Lamm D (2011). A review of current guidelines and best practice recommendations for the management of nonmuscle invasive bladder cancer by the international bladder cancer group. *Journal of Urology*.

[18] de Nunzio C, Carbone A, Albisinni S (2011). Long-term experience with early single Mitomycin C instillations in patients with low-risk non-muscle-invasive bladder cancer: prospective, single-centre randomised trial. *World Journal of Urology*.

[19] Perlis N, Zlotta AR, Beyene J, Finelli A, Fleshner NE, Kulkarni GS (2013). Immediate post-transurethral resection of bladder tumor intravesical chemotherapy prevents non-muscle-invasive bladder cancer recurrences: an updated meta-analysis on 2548 patients and quality-of-evidence review. *European Urology*.

[20] Hou JC-T, Landas S, Wang CY, Shapiro O (2011). Instillation of mitomycin C after transurethral resection of bladder cancer impairs wound healing: an animal model. *Anticancer Research*.

[21] Aagaard MF, Mogensen K, Hermann GG (2013). Delayed healing at transurethral resection of bladder tumour sites after immediate postoperative mitomycin C instillation. *Scandinavian Journal of Urology*.

[22] Tolley DA, Parmar MKB, Grigor KM (1996). The effect of intravesical mitomycin C on recurrence of newly diagnosed superficial bladder cancer: a further report with 7 years of followup. *Journal of Urology*.

[23] Sylvester RJ, Oosterlinck W, van Der Meijden APM (2004). A single immediate postoperative instillation of chemotherapy decreases the risk of recurrence in patients with stage Ta T1 bladder cancer: a meta-analysis of published results of randomized clinical trials. *Journal of Urology*.

[24] Barocas DA, Liu A, Burks FN (2013). Practice based collaboration to improve the use of immediate intravesical therapy after resection of nonmuscle invasive bladder cancer. *The Journal of Urology*.

[25] Birder LA, Ruggieri M, Takeda M (2012). How does the urothelium affect bladder function in health and disease?: ICI-RS 2011. *Neurourology and Urodynamics*.

[26] Costantini E, Lazzeri M, Pistolesi D (2013). Morphological changes of bladder mucosa in patients who underwent instillation with combined sodium hyaluronic Acid-chondroitin sulphate (ialuril(R)). *Urologia Internationalis*.

[27] Bassi PF, Volpe A, D’Agostino D (2011). Paclitaxel-hyaluronic acid for intravesical therapy of bacillus calmette-gurin refractory carcinoma in situ of the bladder: results of a phase i study. *Journal of Urology*.

[28] Apodaca G (2004). The uroepithelium: not just a passive barrier. *Traffic*.

[29] Birder LA, Wolf-Johnston AS, Chib MK, Buffington CA, Roppolo JR, Hanna-Mitchell AT (2010). Beyond neurons: involvement of urothelial and glial cells in bladder function. *Neurourology and Urodynamics*.

[30] Janssen DA, van Wijk XM, Jansen KC, van Kuppevelt TH, Heesakkers JP, Schalken JA (2013). The distribution and function of chondroitin sulfate and other sulfated glycosaminoglycans in the human bladder and their contribution to the protective bladder barrier. *The Journal of Urology*.

[31] Hurst RE, Roy JB, Min KW (1996). A deficit of chondroitin sulfate proteoglycans on the bladder uroepithelium in interstitial cystitis. *Urology*.

[32] Ananthasubramaniam K, Weiss R, McNutt B, Klauke B, Feaheny K, Bukofzer S (2012). A randomized, double-blind, placebo-controlled study of the safety and tolerance of regadenoson in subjects with stage 3 or 4 chronic kidney disease. *Journal of Nuclear Cardiology*.

[33] Geppetti P, Nassini R, Materazzi S, Benemei S (2008). The concept of neurogenic inflammation. *BJU International, Supplement*.

[34] Damiano R, Quarto G, Bava I (2011). Prevention of recurrent urinary tract infections by intravesical administration of hyaluronic acid and chondroitin sulphate: a placebo-controlled randomised trial. *European Urology*.

[35] Birder LA, Ruan HZ, Chopra B (2004). Alterations in P2X and P2Y purinergic receptor expression in urinary bladder from normal cats and cats with interstitial cystitis. *The American Journal of Physiology—Renal Physiology*.

[36] Birder LA, Apodaca G, De Groat WC, Kanai AJ (1998). Adrenergic- and capsaicin-evoked nitric oxide release from urothelium and afferent nerves in urinary bladder. *The American Journal of Physiology—Renal Physiology*.

[37] Chopra B, Barrick SR, Meyers S (2005). Expression and function of bradykinin B1 and B2 receptors in normal and inflamed rat urinary bladder urothelium. *Journal of Physiology*.

[38] Giglio D, Tobin G (2009). Muscarinic receptor subtypes in the lower urinary tract. *Pharmacology*.

[39] Birder LA (2010). Urothelial signaling. *Autonomic Neuroscience: Basic and Clinical*.

[40] Lazzeri M, Costantini E, Porena M (2009). TRP family proteins in the lower urinary tract: translating basic science into new clinical prospective. *Therapeutic Advances in Urology*.

[41] Stein RJ, Santos S, Nagatomi J (2004). Cool (TRPM8) and hot (TRPV1) receptors in the bladder and male genital tract. *Journal of Urology*.

[42] Streng T, Axelsson HE, Hedlund P (2008). Distribution and function of the hydrogen sulfide-sensitive TRPA1 ion channel in rat urinary bladder. *European Urology*.

[43] Andersson K-E, Gratzke C, Hedlund P (2010). The role of the transient receptor potential (TRP) superfamily of cation-selective channels in the management of the overactive bladder. *BJU International*.

[44] Maggi CA, Santicioli P, Geppetti P (1987). The contribution of capsaicin-sensitive innervation to activation of the spinal vesico-vesical reflex in rats: relationship between substance P levels in the urinary bladder and the sensory-efferent function of capsaicin-sensitive sensory neurons. *Brain Research*.

[45] Sculptoreanu A, De Groat WC, Tony Buffington CA, Birder LA (2005). Abnormal excitability in capsaicin-responsive DRG neurons from cats with feline interstitial cystitis. *Experimental Neurology*.

[46] Dinis P, Charrua A, Avelino A (2004). Anandamide-evoked activation of vanilloid receptor 1 contributes to the development of bladder hyperreflexia and nociceptive transmission to spinal dorsal horn neurons in cystitis. *Journal of Neuroscience*.

[47] Lazzeri M, Montorsi F (2011). The therapeutic challenge of “chronic cystitis”: search well, work together, and gain results. *European Urology*.

[48] Carter BD, Kaltschmidt C, Kaltschmidt B (1996). Selective activation of NF-*κ*B by nerve growth factor through the neurotrophin receptor p75. *Science*.

[49] Iavazzo C, Athanasiou S, Pitsouni E, Falagas ME (2007). Hyaluronic acid: an effective alternative treatment of interstitial cystitis, recurrent urinary tract infections, and hemorrhagic cystitis?. *European Urology*.

[50] Montell C, Rubin GM (1989). Molecular characterization of the drosophila trp locus: a putative integral membrane protein required for phototransduction. *Neuron*.

[51] Skryma R, Prevarskaya N, Gkika D, Shuba Y (2011). From urgency to frequency: facts and controversies of TRPs in the lower urinary tract. *Nature Reviews Urology*.

[52] Yu W, Hill WG, Apodaca G, Zeidel ML (2011). Expression and distribution of transient receptor potential (TRP) channels in bladder epithelium. *The American Journal of Physiology—Renal Physiology*.

[53] Everaerts W, Gevaert T, Nilius B, De Ridder D (2008). On the origin of bladder sensing: Tr(i)ps in urology. *Neurourology and Urodynamics*.

[54] Birder L, Andersson KE (2013). Urothelial signaling. *Physiological Reviews*.

[55] Szallasi A, Conte B, Goso C, Blumberg PM, Manzini S (1993). Characterization of a peripheral vanilloid (capsaicin) receptor in the urinary bladder of the rat. *Life Sciences*.

[56] Lazzeri M, Vannucchi MG, Zardo C (2004). Immunohistochemical evidence of vanilloid receptor 1 in normal human urinary bladder. *European Urology*.

[57] Birder LA, Nakamura Y, Kiss S (2002). Altered urinary bladder function in mice lacking the vanilloid receptor TRPV1. *Nature Neuroscience*.

[58] Avelino A, Cruz F (2006). TRPV1 (vanilloid receptor) in the urinary tract: expression, function and clinical applications. *Naunyn-Schmiedeberg’s Archives of Pharmacology*.

[59] Daly D, Rong W, Chess-Williams R, Chapple C, Grundy D (2007). Bladder afferent sensitivity in wild-type and TRPV1 knockout mice. *Journal of Physiology*.

[60] Maggi CA, Barbanti G, Santicioli P (1989). Cystometric evidence that capsaicin-sensitive nerves modulate the afferent branch of micturition reflex in humans. *Journal of Urology*.

[61] Cruz F, Guimarães M, Silva C, Rio ME, Coimbra A, Reis M (1997). Desensitization of bladder sensory fibers by intravesical capsaicin has long lasting clinical and urodynamic effects in patients with hyperactive or hypersensitive bladder dysfunction. *Journal of Urology*.

[62] Payne CK, Mosbaugh PG, Forrest JB (2005). Intravesical resiniferatoxin for the treatment of interstitial cystitis: a randomized, double-blind, placebo controlled trial. *Journal of Urology*.

[63] Kunzelmann K (2005). Ion channels and cancer. *Journal of Membrane Biology*.

[64] Tsavaler L, Shapero MH, Morkowski S, Laus R (2001). Trp-p8, a novel prostate-specific gene, is up-regulated in prostate cancer and other malignancies and shares high homology with transient receptor potential calcium channel proteins. *Cancer Research*.

[65] Fuessel S, Sickert D, Meye A (2003). Multiple tumor marker analyses (PSA, hK2, PSCA, trp-p8) in primary prostate cancers using quantitative RT-PCR. *International Journal of Oncology*.

[66] Fixemer T, Wissenbach U, Flockerzi V, Bonkhoff H (2003). Expression of the Ca^2+^-selective cation channel TRPV6 in human prostate cancer: a novel prognostic marker for tumor progression. *Oncogene*.

[67] Kalogris C, Caprodossi S, Amantini C (2010). Expression of transient receptor potential vanilloid-1 (TRPV1) in urothelial cancers of human bladder: relation to clinicopathological and molecular parameters. *Histopathology*.

[68] Lopez-Carrillo L, Avila MH, Dubrow R (1994). Chili pepper consumption and gastric cancer in Mexico: a case-control study. *The American Journal of Epidemiology*.

[69] Kim SR, Kim SU, Oh U, Jin BK (2006). Transient receptor potential vanilloid subtype 1 mediates microglial cell death in vivo and in vitro via Ca^2+^-mediated mitochondrial damage and cytochrome c release. *Journal of Immunology*.

[70] Amantini C, Mosca M, Nabissi M (2007). Capsaicin-induced apoptosis of glioma cells is mediated by TRPV1 vanilloid receptor and requires p38 MAPK activation. *Journal of Neurochemistry*.

[71] Shin D-H, Kim O-H, Jun H-S, Kang M-K (2008). Inhibitory effect of capsaicin on B16-F10 melanoma cell migration via the phosphatidylinositol 3-kinase/Akt/Rac1 signal pathway. *Experimental and Molecular Medicine*.

[72] Vinuesa AG, Sancho R, García-Limones C (2012). Vanilloid receptor-1 regulates neurogenic inflammation in colon and protects mice from colon cancer. *Cancer Research*.

[73] Lazzeri M, Vannucchi MG, Spinelli M (2005). Transient receptor potential vanilloid type 1 (TRPV1) expression changes from normal urothelium to transitional cell carcinoma of human bladder. *European Urology*.

[74] Amantini C, Ballarini P, Caprodossi S (2009). Triggering of transient receptor potential vanilloid type 1 (TRPV1) by capsaicin induces Fas/CD95-mediated apoptosis of urothelial cancer cells in an ATM-dependent manner. *Carcinogenesis*.

[75] Caprodossi S, Amantini C, Nabissi M (2011). Capsaicin promotes a more aggressive gene expression phenotype and invasiveness in null-TRPV1 urothelial cancer cells. *Carcinogenesis*.

[76] Surh Y-J (2002). More than spice: capsaicin in hot chili peppers makes tumor cells commit suicide. *Journal of the National Cancer Institute*.

[77] Qiao S, Li W, Tsubouchi R, Haneda M, Murakami K, Yoshino M (2005). Involvement of peroxynitrite in capsaicin-induced apoptosis of C6 glioma cells. *Neuroscience Research*.

[78] Gonzales CB, Kirma NB, De La Chapa JJ (2014). Vanilloids induce oral cancer apoptosis independent of TRPV1. *Oral Oncology*.

[79] Itokawa H, Shi Q, Akiyama T, Morris-Natschke SL, Lee K-H (2008). Recent advances in the investigation of curcuminoids. *Chinese Medicine*.

[80] Pan M-H, Huang T-M, Lin J-K (1999). Biotransformation of curcumin through reduction and glucuronidation in mice. *Drug Metabolism and Disposition*.

[81] Osawa T, Sugiyama Y, Inayoshi M, Kawakishi S (1995). Antioxidative activity of tetrahydrocurcuminoids. *Bioscience, Biotechnology, and Biochemistry*.

[82] Sugiyama Y, Kawakishi S, Osawa T (1996). Involvement of the *β*-diketone moiety in the antioxidative mechanism of tetrahydrocurcumin. *Biochemical Pharmacology*.

[83] Lai C-S, Wu J-C, Yu S-F (2011). Tetrahydrocurcumin is more effective than curcumin in preventing azoxymethane-induced colon carcinogenesis. *Molecular Nutrition and Food Research*.

[84] Shehzad A, Wahid F, Lee YS (2010). Curcumin in cancer chemoprevention: molecular targets, pharmacokinetics, bioavailability, and clinical trials. *Archiv der Pharmazie*.

[85] Shehzad A, Rehman G, Lee YS (2013). Curcumin in inflammatory diseases. *Biofactors*.

[86] Cheng T-C, Lu C-C, Chung H-H (2010). Activation of muscarinic M-1 cholinoceptors by curcumin to increase contractility in urinary bladder isolated from Wistar rats. *Neuroscience Letters*.

[87] Arafa HMM (2009). Uroprotective effects of curcumin in cyclophosphamide-induced haemorrhagic cystitis paradigm. *Basic and Clinical Pharmacology and Toxicology*.

[88] Lu Q, Jiang F, Xu R (2013). A pilot study on intravesical administration of curcumin for cystitis glandularis. *Evidence-Based Complementary and Alternative Medicine*.

[89] Olivera A, Moore TW, Hu F (2012). Inhibition of the NF-*κ*B signaling pathway by the curcumin analog, 3,5-Bis(2-pyridinylmethylidene)-4-piperidone (EF31): anti-inflammatory and anti-cancer properties. *International Immunopharmacology*.

[90] Dorai T, Gehani N, Katz A (2000). Therapeutic potential of curcumin in human prostate cancer - I. Curcumin induces apoptosis in both androgen-dependent and androgen-independent prostate cancer cells. *Prostate Cancer and Prostatic Diseases*.

[91] Cimino S, Sortino G, Favilla V (2012). Polyphenols: key issues involved in chemoprevention of prostate cancer. *Oxidative Medicine and Cellular Longevity*.

[92] Sundram V, Chauhan SC, Ebeling M, Jaggi M (2012). Curcumin attenuates *β*-catenin signaling in prostate cancer cells through activation of protein kinase D1. *PLoS ONE*.

[93] Capodice JL, Gorroochurn P, Cammack AS (2009). Zyflamend in men with high-grade prostatic intraepithelial neoplasia: results of a phase I clinical trial. *Journal of the Society for Integrative Oncology*.

[94] Tian B, Wang Z, Zhao Y (2008). Effects of curcumin on bladder cancer cells and development of urothelial tumors in a rat bladder carcinogenesis model. *Cancer Letters*.

[95] Chadalapaka G, Jutooru I, Chintharlapalli S (2008). Curcumin decreases specificity protein expression in bladder cancer cells. *Cancer Research*.

[96] Watanabe FT, Chade DC, Reis ST (2011). Curcumin, but not Prima-1, decreased tumor cell proliferation in the syngeneic murine orthotopic bladder tumor model. *Clinics*.

[97] Tharakan ST, Inamoto T, Sung B, Aggarwal BB, Kamat AM (2010). Curcumin potentiates the antitumor effects of gemcitabine in an orthotopic model of human bladder cancer through suppression of proliferative and angiogenic biomarkers. *Biochemical Pharmacology*.

[98] Martelli L, Ragazzi E, Di Mario F (2007). A potential role for the vanilloid receptor TRPV1 in the therapeutic effect of curcumin in dinitrobenzene sulphonic acid-induced colitis in mice. *Neurogastroenterology and Motility*.

[99] Sharma S, Kulkarni SK, Agrewala JN, Chopra K (2006). Curcumin attenuates thermal hyperalgesia in a diabetic mouse model of neuropathic pain. *European Journal of Pharmacology*.

[100] Mittal N, Joshi R, Hota D, Chakrabarti A (2009). Evaluation of antihyperalgesic effect of curcumin on formalin-induced orofacial pain in rat. *Phytotherapy Research*.

[101] Yeon KY, Kim SA, Kim YH (2010). Curcumin produces an antihyperalgesic effect via antagonism of TRPV1. *Journal of Dental Research*.

[102] Gallimore AM, Godkin A (2013). Epithelial barriers, microbiota, and colorectal cancer. *The New England Journal of Medicine*.

